# A Rare Case of Malignant Triton Tumor Without Associated Neurofibromatosis

**DOI:** 10.7759/cureus.69016

**Published:** 2024-09-09

**Authors:** Hriday Shah, Prutha Tailor, Khushbu Shah, Twinkle Shah

**Affiliations:** 1 Internal Medicine, St. Clare's Denville Hospital, Denville, USA; 2 Medicine, Gujarat Medical Education and Research Society (GMERS) Gotri Medical College, Vadodara, IND; 3 Pathology, Gujarat Medical Education and Research Society (GMERS) Gotri Medical College, Vadodara, IND; 4 Medicine, Gujarat Medical Education and Research Society (GMERS) Medical College and Hospital Valsad, Valsad, IND

**Keywords:** desmin positive, malignant triton tumor (mtt), neurofibromatosis type 1 (nf-1), nf-1, oral carcinoma, recurrent tumor, rhabdomyoblastic differentiation, s100, von recklinghausen disease

## Abstract

This paper describes a rare case of malignant triton tumor (MTT), a malignant peripheral nerve sheath tumor with rhabdomyoblastic differentiation, that occurred in a 21-year-old male with no concomitant clinical signs of neurofibromatosis. Although total surgical excision is ideal, the high recurrence rate and distant metastases frequently result in a poor prognosis. A biopsy, in this case, revealed spindle cells organized in short fascicles, with minor anisonucleosis and cross-striations indicating rhabdomyomatous differentiation. Positive immunohistochemistry for epithelial membrane antigen (EMA), Desmin, and S100 markers validated the diagnosis. For further treatment, the patient was referred to a cancer center. Even without clinical signs of neurofibromatosis type 1 (NF-1), the research emphasizes the need to evaluate MTTs as a differential diagnosis for individuals presenting with tumors in the head and neck area. Histopathology and immunohistochemistry are useful diagnostic techniques, and early intervention with surgical excision may enhance the outcome. The paper concludes with a review of the histological criteria needed to establish the diagnosis of MTT and the distinctions between sporadic and NF-1-associated tumors.

## Introduction

Malignant triton tumor (MTT, also known as malignant peripheral nerve sheath tumor with rhabdomyoblastic differentiation) is a rare disease with an unfavorable prognosis. The tumor is linked with von Recklinghausen neurofibromatosis (NF-1) in 70% of cases and is the only morbid finding in the remaining 30%. Due to a diverse histopathological presentation, it places great importance not only on the clinical history but also on various differential diagnoses. Schwann cells, originally arising from neural crest cells, retain their capacity for mesenchymal differentiation (most commonly into skeletal muscle) during malignant transformation. Local recurrence is common, with distant metastasis primarily occurring in the lung and brain. Surgery is the preferred treatment, and post-operative radiation is always recommended. Histopathology and immunohistochemistry can assist in the diagnosis [1]. People with initial nose and paranasal sinus tumors have a better prognosis, but individuals with primary neck tumors have a worse prognosis. Every other location shows an intermediate course. Complete surgical removal is essential. Further radiation or chemotherapy shows little effect [2]. Here, we present a rare case of MTT of the buccal mucosa in a 21-year-old male with no associated clinical features of neurofibromatosis.

## Case presentation

**Figure 1:**
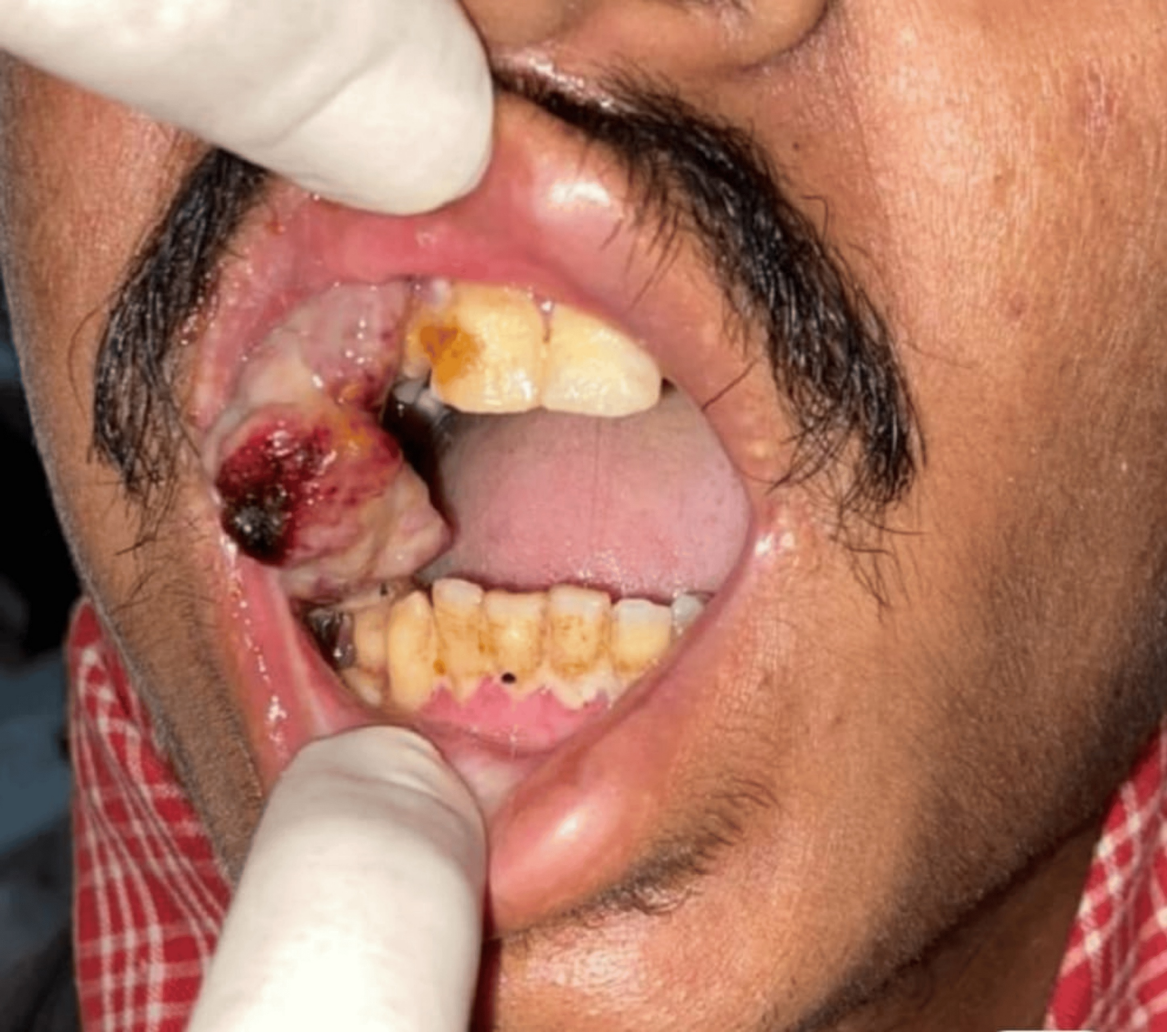
Initial presentation of swelling.

A 21-year-old male presented with a progressively increasing mass over the right buccal mucosa for six months (Figure 1). The mass was firm, tender, and bled to the touch on physical examination. It measured 3 x 2 cm², located in the right buccal mucosa near the first premolar and extending up to the right angle of the mandible. There were no enlarged neck lymph nodes and no history of significant weight loss. The rest of the examination was normal. The patient's family history was insignificant. He did not have any café-au-lait spots, freckles, or neurofibromas.

The patient was admitted; he was vitally stable, and his blood and urinalysis investigations were normal. Following the above investigation, the patient underwent resection of the mass. The resected mass biopsy was sent to the lab for histopathological examination. The diagnosis of benign inflammatory myofibroblastic proliferation was made, and the patient was discharged on symptomatic treatment.

**Figure 2:**
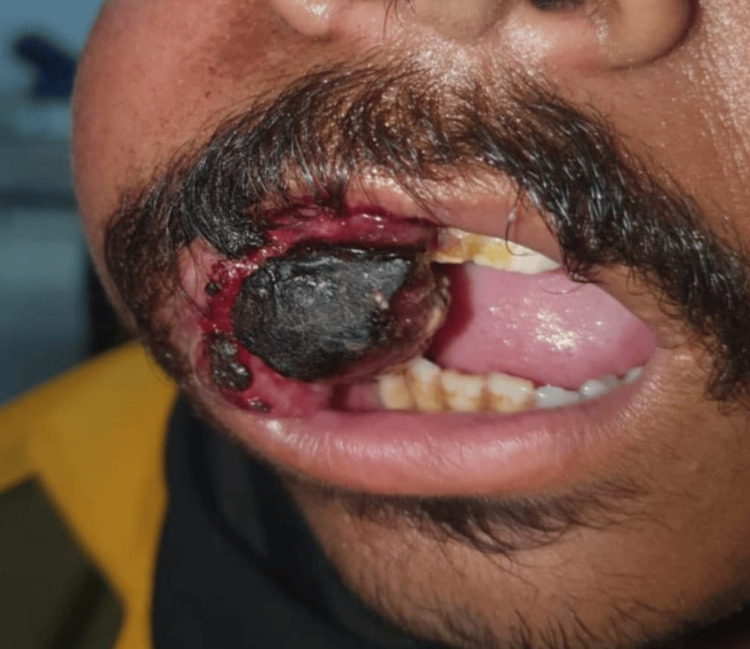
Swelling observed during the latest visit.

**Figure 3:**
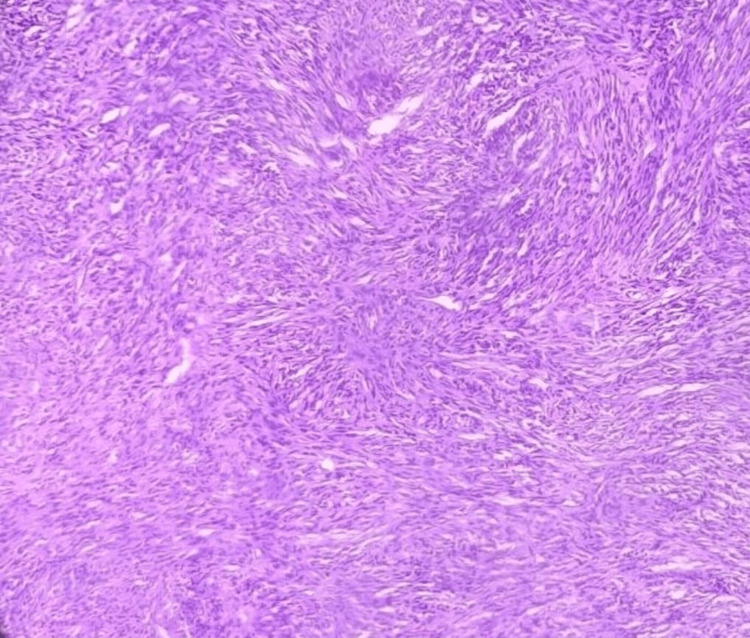
High-power H&E stained image.

After three months, the patient again presented with a similar but larger mass at the exact same location (Figure 2). The mass was 5 x 4 cm², extending from the right buccal mucosa to the retromandibular space. It was firm and tender, with bleeding on touch and areas of necrosis present. He was admitted, and a biopsy was sent for histopathology and immunohistochemistry. Histopathology showed mainly spindle cells arranged in short fascicles (Figure 3). The cells were spindle-shaped, having spindle to oval normochromic nuclei, and mild anisonucleosis was observed. Cross-striations indicated the possibility of rhabdomyomatous differentiation. On immunohistochemistry examination, markers epithelial membrane antigen (EMA), Desmin, and S100 were positive, suggesting an MTT.

The patient was referred to the oncology center for further management.

## Discussion

Malignant schwannoma with rhabdomyoblastic differentiation is a rare cancer, also known as a MTT. Woodruff coined the term 'malignant triton tumor' to characterize such tumors [3]. Woodruff published the original histological criteria for establishing the diagnosis of MTT in 1973. According to these criteria, the tumor must arise along the course of a peripheral nerve in a patient with NF-1 (Neurofibromatosis-1), display most of the growth characteristics of Schwann cells, and contain bona fide rhabdomyoblasts [3]. There are two types of tumors: sporadic and NF-1-associated. Around 70% have von Recklinghausen neurofibromatosis, with a male predominance, early age, and a frequent presentation in the head and neck. Those without von Recklinghausen neurofibromatosis, on the other hand, are more prevalent at a later age, have a female predominance, and are usually seen on the trunk [1]. However, in our case, a young male with an MTT had no clinical features of NF-1. Histopathology can be used to diagnose MTTs if the S-100 protein is positive. Most regions display the appearance of a very cellular spindle cell neoplasm with several mitoses in morphology. Although most tumors are monomorphic, heterologous components such as rhabdomyoblasts, cartilage, and bone may be present in rare situations. S-100 protein positivity in 50-90% of these tumors indicates a nerve sheath origin. Desmin, myogenin, and MyoD1 immunostains are positive in rhabdomyoblasts [4]. On immunohistochemistry, our case was positive for S-100, Desmin, and EMA (E29). Histologically, our case showed mainly spindle cells arranged in short fascicles. Cells were spindle in shape, having spindle to oval normochromic nuclei, and mild anisonucleosis was observed. There is no consensus on treatment protocols for MTTs. Many authors have advocated various recommendations, including radical excision followed by radiation and chemotherapy or excision plus radiotherapy. Nonetheless, as with any other sarcoma, excision of the tumor with broad margins followed by radiation is the recommended treatment, with no evident need for chemotherapy [3]. MTT has a poor prognosis, with a 5-15% five-year survival rate depending on the location, grade, and completeness of radical resection. The prognosis is favorable in the head, neck, and extremities, but it is very poor in the buttocks and other areas. Despite effective therapy, local recurrence and distant metastasis rates are around 25% and 48%, respectively. Patients with head and neck MTT had a survival range of four months to 22 years. Cytogenetic analysis of this tumor revealed several karyotypic alterations. Cytogenetic studies have identified certain breakpoints that are thought to be regions for myogenic differentiation and are likely involved in rhabdomyoblastic differentiation [1].

## Conclusions

MTTs are rare tumors with a poor prognosis. They are mostly associated with NF-1, and sporadic cases are rare. Diagnosis is difficult and necessitates histological and immunohistochemical investigation. Surgery with post-operative radiation is the primary therapy. We describe a rare case of MTT of the buccal mucosa in a 21-year-old man with no clinical symptoms of neurofibromatosis. MTTs should be considered in young individuals with tumors that display rhabdomyoblastic differentiation, even if NF-1 is absent.
